# Dietary phytochemicals and neuro-inflammaging: from mechanistic insights to translational challenges

**DOI:** 10.1186/s12979-016-0070-3

**Published:** 2016-04-14

**Authors:** Sergio Davinelli, Michael Maes, Graziamaria Corbi, Armando Zarrelli, Donald Craig Willcox, Giovanni Scapagnini

**Affiliations:** Department of Medicine and Health Sciences, School of Medicine, University of Molise, Campobasso, Italy; IMPACT Research Center, Deakin University, Geelong, Australia; Department of Psychiatry, Faculty of Medicine, Chulalongkorn University, Bangkok, Thailand; Department of Chemical Sciences, University of Naples “Federico II”, Complesso Universitario Monte S. Angelo, Naples, Italy; Department of Human Welfare, Okinawa International University, Okinawa, Japan; Department of Geriatric Medicine, John A. Burns School of Medicine, University of Hawaii, Honolulu, USA

**Keywords:** Brain, Aging, Diet, Phytochemicals, Inflammation, Oxidative stress

## Abstract

An extensive literature describes the positive impact of dietary phytochemicals on overall health and longevity. Dietary phytochemicals include a large group of non-nutrients compounds from a wide range of plant-derived foods and chemical classes. Over the last decade, remarkable progress has been made to realize that oxidative and nitrosative stress (O&NS) and chronic, low-grade inflammation are major risk factors underlying brain aging. Accumulated data strongly suggest that phytochemicals from fruits, vegetables, herbs, and spices may exert relevant negative immunoregulatory, and/or anti-O&NS activities in the context of brain aging. Despite the translational gap between basic and clinical research, the current understanding of the molecular interactions between phytochemicals and immune-inflammatory and O&NS (IO&NS) pathways could help in designing effective nutritional strategies to delay brain aging and improve cognitive function. This review attempts to summarise recent evidence indicating that specific phytochemicals may act as positive modulators of IO&NS pathways by attenuating pro-inflammatory pathways associated with the age-related redox imbalance that occurs in brain aging. We will also discuss the need to initiate long-term nutrition intervention studies in healthy subjects. Hence, we will highlight crucial aspects that require further study to determine effective physiological concentrations and explore the real impact of dietary phytochemicals in preserving brain health before the onset of symptoms leading to cognitive decline and inflammatory neurodegeneration.

## Background

Over the next few decades, given the rising life expectancy within the older population, the incidence of developing age-related neurodegenerative diseases is predicted to increase dramatically. A critical factor that plays a crucial role in brain aging is the exceptionally high energy demand of neurons to preserve neuronal processes and maintain cognitive ability. The high consumption of oxygen for the generation of energy may reflect more vulnerability of the brain to reactive species attack and subsequent inflammation [1]. In addition, normal brain metabolism is associated with more than one process contributing to functional impairment in brain aging. For instance, high content of polyunsaturated fatty acids (PUFAs) in neuronal membranes (easily peroxidizable), limited amount of endogenous antioxidant defences, low rate of cell turnover, and high production of reactive oxygen and nitrogen species (ROS/RNS) put the brain at risk to oxidative and nitrosative (O&NS) damage [2]. It should be also mentioned that the age-related redox imbalance underlying the pathophysiological basis of neurodegeneration is accompanied by many types of cellular damages, including oxidative DNA and lipid damage, oxidative and nitrosative protein damage, mitochondrial damage, telomere attrition and accumulation of macromolecular waste [2, 3]. Growing studies suggest that a bidirectional communication between brain and immune system is crucial to maintain central nervous system (CNS) homeostasis. To date, one of most recognized effects of brain aging is the dysregulation of the immune system as a result of uncontrolled production of reactive species and pro-inflammatory cytokines [4, 5]. This chronic condition has been defined as “oxi-inflammaging” and may contribute to neuronal loss in different neurodegenerative diseases, culminating in accelerated neurodegeneration [6–9]. During brain aging, the generation of ROS and RNS increases the synthesis of numerous chemokines and pro-inflammatory cytokines, including interleukin (IL)-1, IL-6, and tumor necrosis factor- α (TNF-α). These inflammatory mediators activate microglia and astrocytes to generate large amounts of ROS/RNS. Recent studies showed that neuroinflammatory processes may be considered as a consequence of chronic oxidative stress [10, 11]. Moreover, the neuropathological alterations associated with oxidative stress and pro-inflammatory state include deposition of insoluble materials such as amyloid-β (Aβ) plaques and neurofibrillary tangles (NFTs), which are the main factors of cell death and age-related dementia [12].

However, it is now becoming clear that the consumption of diets rich in phytochemicals can influence neuroinflammation and mediate the activation of signaling pathways, leading to the expression of cytoprotective and restorative proteins [13, 14]. There is substantial evidence supporting the notion that bioactive dietary components may contribute to develop novel therapies capable of preventing the progressive dysfunction of neuronal populations that underlie neurodegeneration [15, 16]. A large variety of foods including fruit, vegetables, cereals, nuts and cocoa/chocolate as well as tea, coffee and wine contain a wide range of plant secondary metabolites, better known as phytochemicals, which have been shown to be effective in increasing antioxidant enzymes, neurotrophic factors and anti-apoptotic proteins. Several mechanisms have been proposed for the health benefits of phytochemicals, however, their ability to modulate signal transduction cascades and activate transcription factors that antagonize neuroinflammation and O&NS has attracted considerable interest [17]. Despite various beneficial biological activities, phytochemicals may have carcinogenic or genotoxic effects at high doses or concentrations [18]. Therefore, the challenge is to establish the exact dose range and perform human intervention studies to develop effective nutritional strategies capable of counteracting neuroinflammatory processes that accompany brain aging. Here, we discuss some of the new findings that provide insight into how phytochemicals positively affect neuroinflammation and brain aging. Specifically, we will discuss the main neuroprotective activities of phytochemicals that have been studied in cells, animals and humans emphasizing the importance of dosage, which is crucial to establish endpoints for clinical studies and develop dietary recommendations.

## Oxidative stress, microglial redox activation and neuroinflammation

Aging is associated with an imbalance in redox status in a variety of cells and tissues, including the brain. Increasing O&NS stress is thought to be one of the main aging processes causing direct cell loss and cell damage within the brain architecture [19–21]. An age-associated increase in O&NS damage has been shown in neurons of human and rodent brains, and a selective susceptibility of different neuronal populations to oxidative stress has been demonstrated. For instance, oligodendrocytes are particularly vulnerable to oxidative activity due to their role in myelin maintenance and production and limited repair mechanisms [22]. Neuronal oxidation can lead to the destruction of subcellular structures and membranes. Indeed, during brain aging, in addition to the impaired function of many intracellular components such as mitochondrial electron transport chain, various important classes of macromolecules are particularly liable to the deleterious effects of oxidative modification. Several studies have indicated that oxidative damage to nucleic acids can actively contributes to the background, onset, and development of neurodegenerative disorders [23]. Age-associated accumulation of oxidative DNA damage such as the presence of the modified base 8-hydroxydeoxyguanosine (8-OHdG), was observed in many neuron types, including cerebellar granule cells, retinal ganglion, and amacrine, and horizontal cells [24]. Interestingly, it has been recently demonstrated in human neurons that oxidative RNA modification can occur not only in protein-coding RNAs but also in non-coding RNAs, leading to activate inappropriate cell fate pathways [23]. Oxidative damage of the brain is also characterized by increased lipid peroxidation and it has been shown that redox changes in membrane fatty acid composition contribute to the deterioration of neuronal functions. PUFAs, such as arachidonic acid (AA), are abundant in the brain and are highly susceptible to free radical attack during brain aging [25].

**Fig. 1:**
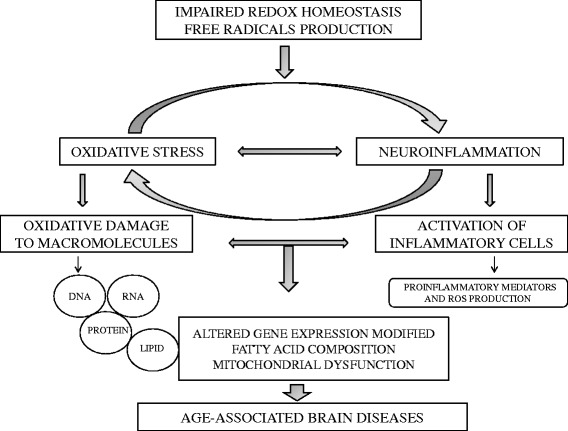
Oxidative stress, chronic inflammation and brain aging are closely linked. The figure depicts the central roles of free radicals linking oxidative stress to inflammation and brain aging

Hypernitrosylation may inhibit the functions of many different proteins involved in critical cell functions leading to apoptosis, dysfunctions in intracellular signalling, inhibition of cell growth, mitochondrial functions, cell death, etc. [20]. Another consequence of nitrosative stress is the formation of immunogenic neoepitopes, e.g. nitroso (NO)-tyrosine and NO-tryptophan which may mount autoimmune responses, e.g. IgM-mediated autoimmune responses to NO-cysteinyl [20]. The latter autoimmune responses may have neurotoxic effects and cause demyelination. Hypernitrosylation is tightly coupled to nuclear factor (NF)- κB activation which induces and supports inflammaging and induction of inducible NO-synthase (iNOS). An age-related NF-κB constitutive activation was observed in many tissues including brain [26]. Increased nitration of proteins, e.g. the formation of immunogenic nitro (NO2)-tyrosine, accompanies the aging process [27]. Besides oxidation of nucleic acids, lipids, and proteins, and nitrosylation, nitrosation and nitration of proteins, an excess of free radical generation is associated with chronic low-grade inflammation and the onset of age-related brain disorders [28]. The role of an excessive free radical production linked to persistent low-basal inflammation and brain aging is schematized in Fig. 1. In the CNS, reactive species, glial cells and inflammatory mediators form a coordinated network to maintain a proper equilibrium between physiological and pathological processes. In the brain, glial cells (mainly microglia) provide the first line of defense against noxious agents or injurious processes [29]. Microglial cells are the resident macrophages of the CNS and under physiological conditions they exhibit a deactivated phenotype. A tight control of microglial inflammatory response is essential for CNS homeostasis to promote the clearance of pathogens, toxic cellular debris and stimulate repair processes after brain damage. However, microglia are a potent cellular source of oxidation products and inflammatory molecules during brain aging. Microglia respond to oxidative damage, as a result of aging or neurodegeneration, by releasing a large spectrum of neuroactive mediators. The accumulation of oxidative damage in microglia during aging results in the increased production of ROS. Disproportional increase in intracellular ROS can activate the redox-sensitive NF-κB and provoke excessive neuroinflammation [30]. A sustained activation of microglia may lead to neuronal degeneration and dysfunction, as observed in brain aging, cognitive and neuropsychiatric disorders [31]. An interruption in microglial homeostasis, a process referred to as microglial priming, makes the microglia more susceptible to inflammatory stimuli and neurodegeneration and may be an early events leading to oxidative damage and depletion of endogenous antioxidants [32]. The causal relationship between oxidative damage and neuroinflammatory response is exemplified by the cytokine TNF-α, which is released by activated microglia. Although TNF-α can ameliorate immune responses and promote neuroprotection, under uncontrolled conditions TNF-α is a powerful pro-inflammatory and neurotoxic molecule. In particular, TNF-α can contribute to neuroinflammation by activating the transcription factor NF-κB in glia cells, but also promoting the generation and release of ROS through the NADPH oxidase system and RNS [4, 33, 34]. In addition, recent findings suggest that the dysregulation of Toll-Tike receptors (TLRs) participate to generate a hyperactivated state of microglia. A persistent activation of TLRs, their signalling pathways and downstream effector molecules, may contribute to cytotoxic compounds accumulation such as ROS, RNS, cytokines, complements and proteases causing neuronal loss and damage [35, 36]. The formation of redox-derived damage-associated molecular patterns (DAMPs), may further activate the TLR4 complex leading to a chronic activation of the TLR-radical cycle, thereby causing chronic IO&NS [37]. Additional pro-inflammatory markers, including interleukin (IL)-6, C-reactive protein (CRP), matrix metalloproteinases (MMPs), cytosolic phospholipase A2 (cPLA2), cyclooxygenase-2 (COX-2) and TNF-α are consistently elevated in neurodegenerative diseases, which are largely associated with chronically elevated levels of ROS [5, 38]. Moreover, it has been hypothesized that IL-6 is a central regulator of the neuroinflammatory responses in brain aging. Animal models and patients with neurodegenerative diseases had higher levels of IL-6 and CRP, providing evidence that peripheral inflammatory mediators can increase ROS production and also interfere with neurocognitive functions [39–41]. There is growing interest in the redox pathophysiology of neuroinflammation and emerging concepts open new ways to restore the inactive state of microglia and modulate the low-grade chronic neuroinflammation that characterizes brain aging.

## Neuroprotective signaling pathways

Dietary phytochemicals exert their beneficial effects on the nervous system modulating cellular stress response signaling pathways [42]. At low doses, several phytochemicals are known to enhance neuronal stress resistance, whereas at high doses many different phytochemicals can be toxic. This is an example of biphasic dose–response relationship with stimulatory or beneficial effects at low doses and inhibitory or adverse effects at high doses (hormesis) [43]. Therefore, dietary phytochemicals act as mild stressors to induce adaptive expression of stress-protective genes in neuronal cells and enhance resistance to the mechanisms that determine brain aging. In the next sections, we highlight the major signaling pathways by which dietary phytochemicals offer neuroprotection in an exposure-related manner.

### The Nrf2 antioxidant response pathway

The transcription factor nuclear factor E2-related factor 2 (Nrf2) has emerged as a key cytoprotective regulator against O&NS stress and neuroinflammation [44]. Nrf2 is a member of the cap‘n’collar (CNC) family of stress-sensing transcription factors. Nrf2 is a basic leucine zipper protein that in the nucleus heterodimerizes with small Maf protein followed by binding to specific DNA sites termed antioxidant response elements (AREs) or electrophile response elements (EpRE) [45]. In basal conditions, Nrf2 is kept transcriptionally inactive and sequestered in the cytoplasm by its repressor protein, the Kelch-like ECH-associated protein 1 (Keap1). This binding provides the turnover of Nrf2 through proteasomal degradation. Keap1, a sulfhydryl-rich protein, is a specialized sensor to quantify stress in presence of oxidative stressors and electrophilic xenobiotics. In response to oxidative and/or electrophilic stress, Nrf2-Keap1 dissociation is triggered with consequent translocation of Nrf2 to the nucleus, where the formed heterodimer with Maf binds ARE sequence in the promoter regions of genes involved in phase II detoxification and antioxidant defense [46]. Nrf2 is ubiquitously present in all tissues but is widely expressed in the CNS where it promotes cell survival and coordinates the transcription of neuroprotective proteins. Some examples include various superoxide dismutase (SOD) isoforms, catalase (CAT), glutathione peroxidase, glutathione reductase, various glutathione-S-transferase (GST) isoforms, NAD(P)H:quinone oxidoreductase 1 (NQO1), and heme oxigenase-1 (HO-1). However, recent findings have linked the activation of the Nrf2 signaling not only to phase II detoxifying enzymes and antioxidants proteins but also to expression of other cytoprotective proteins such as the growth factor brain-derived neurotrophic factor (BDNF), the anti-inflammatory IL-10, the mitochondrial transcription (co)-factors Nrf-1 and peroxisome proliferator-activated receptor gamma coactivator 1-alpha (PGC-1α) [44]. Aging is associated with Nrf2 dysfunction and a dysregulation of this pathway has been linked to the pathogenesis of several chronic disorders, including neurodegenerative, cardiovascular, metabolic, infectious, and pulmonary diseases as well as chronic inflammatory conditions [47–49]. Accumulating evidence suggests that the decline in the Nrf2 signaling system plays a key role in the accumulation of pro-inflammatory mediators and oxidative damage during brain aging [50, 51]. Although the underlying mechanisms behind the impairment of Nrf2 pathway in neurodegeneration are not known, elevated levels of oxidative stress and inflammation in patients with neurodegenerative diseases have been reported. Studies have established that the nuclear translocation of Nrf2 is reduced in the hippocampus of Alzheimer’s disease (AD) patients. Also mRNA and protein levels of Nrf2 are reduced in the motor cortex and spinal cord of amyotrophic lateral sclerosis (ALS) patients [52]. It appears also from studies with genetic deletion of Nrf2 that the absence of Nrf2 may be detrimental during brain aging and contribute to neurodegeneration. However, there are several promising compounds that at least in vitro are able to restore Nrf2 and induce neuronal protection, as discussed below. Indeed, recent findings suggest that Nrf2 may be a therapeutic target for the alleviation of neuroinflammation associated with neurodegeneration [53].

### The NF-κB signaling pathway in brain inflammation

**Fig. 2:**
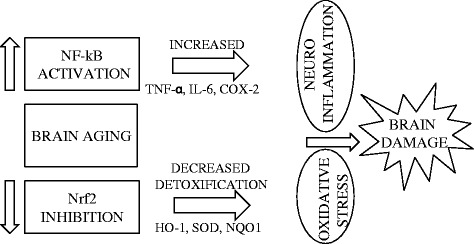
Schematic illustration on the role of Nrf2–NF-κB axis in bran aging. An imbalance between Nrf2 and NF-κB can lead to increased levels of oxidative stress and neuroinflammation, resulting in structural and functional damage to nervous tissue

NF-κB is a ubiquitous stress response pathway and one of the well-described transcription signaling mechanisms. NF-κB transcriptional activity has been associated with neuronal plasticity and neurodegeneration [54]. Indeed, NF-κB is a crucial signal transducer for maintaining CNS homeostasis, particularly in neuronal and glial cells. In response to a wide range of biological stimuli, NF-κB coordinates the expression of numerous genes, encoding pro-inflammatory cytokines, chemokines, and inducible growth factors [55]. The number of target genes of NF-κB is continuously expanding and the deregulation of NF-κB signaling has been detected in multiple disease states, including neurodegenerative diseases and chronic inflammatory conditions. NF-κB orchestrates a broad range of physiological stimuli through canonical and non-canonical pathways. In CNS, NF-κB exists in a latent and a constitutively active form. Constitutively activated NF-κB is mostly detected within the nucleus of glutamatergic neurons and is regulated by synaptic activity. In glia, NF-κB has a lower basal activity and is heavily inducible [56]. Although NF-κB signalling is tightly regulated at multiple levels, in most unstressed cells is located in the cytoplasm in an inactive complex consisting of two subunits of 50 kDa (p50) and 65 kDa (p65) and an inhibitory subunit called IkB (IkBα or IkBβ). In response to activating stimuli, IkB is phosphorylated, ubiquitinated and degraded by the proteasome, which in turn allows the nuclear translocation of NF-κB to regulate gene expression. The most fundamental aspects of NF-κB regulation in the brain have been extensively discussed elsewhere [57–61]. Given that a fundamental goal for future studies is to determine whether activation of NF-κB is important for neuroprotection, in the present paragraph, we would like to highlight some data to demonstrate that NF-κB may be an activator of neuroprotective programs. Neuroprotective effects of NF-κB have been described in several experimental models, indicating the potential protective value of this essential transcription factors in the treatment of neuroinflammation and neurodegeneration [62]. Despite this, the role of NF-κB in mechanisms of brain tolerance is complex because it is involved in both protective and damaging pathways. NF-κB supports neuronal survival by increasing the expression of antioxidants, growth factors, and anti-apoptotic molecules. In contrast, glial NF-κB activation promotes neuronal death by inducing production of pro-inflammatory cytokines [63, 64]. However, it is noteworthy to mention that NF-κB signaling pathway has been identified as one of the major neuroprotective mechanism against AD [62]. The dual role of NF-κB in neuronal death and survival is intriguing but we still have limited knowledge on the molecular details underlying its actions as activator of neuroprotective programs or inducer of neurodegenerative processes. Understanding which determinants are implicated in switching NF-κB from a neuroprotective to a neurotoxic activity may help the development of new treatments for neuroinflammation. In this context, preconditioning/hormesis has been therapeutically linked to activation of NF-κB and inhibition of neuronal apoptosis [65, 66]. Therefore, a low dose of a toxic agent may trigger an adaptive stress-response program mediated by NF-κB and provide neuroprotection. Conversely, high doses of noxious compounds can exacerbate NF-κB activity and induce a neurotoxic response [60, 67]. Furthermore, experimental studies have also demonstrated that neuronal response to external stimuli is dependent on a differential activation of NF-κB dimers. Neurotoxic stimuli or the absence of NF-κB p50 depletes neuronal pro-survival gene products, including anti-apoptotic proteins [68, 69]. Finally, it is remarkable to mention that the identification of NF-κB binding sites in the promoter region of the Nrf2 gene suggests a potential relationship between these two transcription factors [70]. Moreover, it has been demonstrated that HO-1 induced by the Nrf2 inhibits the NF-κB transcriptional apparatus, and thereby HO-1 is one of the key mediator for the interplay between Nrf2 and NF-κB [71]. As already indicated above, increased NF-κB activity during brain aging is associated with enhanced production of pro-inflammatory cytokines such as IL-6, TNF-α, and COX-2. Reduced Nrf2 activity is characterized by decline in HO-1, SOD, and NQO1 leading to increased levels of oxidative stress and neuroinflammation. A schematic illustration of the cross-talk between Nrf2 and NF-κB in the brain is depicted in Fig. 2.

### MAPK signal transduction and its role in brain aging

Mitogen-activated protein kinases (MAPKs) belong to the super-family of serine/threonine kinases that mediate a wide range of cellular responses. MAPKs have emerged as critical players that connect various extracellular signals into intracellular response. Based on the degree of sequence homology, MAPK transduction cascades are organised into at least three subfamilies: the extracellular signal regulated kinases (ERKs), the stress activated protein kinase/c-Jun N terminal kinase (JNK), and the p38 MAPK [72]. These kinases are involved in both survival and death pathways in response to different stresses to regulate cellular processes such as cell proliferation, survival, differentiation, and metabolism. The role of ERKs is usually associated with pro-survival signaling, however, depending on the type and function of a particular neuron, activation of ERKs can either be neuroprotective or can promote cell death [73]. For instance, in vivo studies of neurons derived from the hippocampal tissue revealed that BDNF regulates synapse formation and plasticity by a mechanism involving ERKs, Nrf2 and forkhead box O (FoxO) transcription factors [74]. Furthermore, ERK activation is required for consolidation and reconsolidation of hippocampal-dependent memory [75]. Dysregulation of ERK signaling has been implicated in both neuropsychiatric and neurodegenerative disorders including, schizophrenia, Huntington’s disease (HD), Parkinson’s disease (PD), and AD [76–78]. JNK and p38 are well-known stress-activated MAPKs, because are potently activated by stress signals such as UV light, inflammatory cytokines, and DNA damaging agents. In particular, a large body of evidence indicates that JNK is a key regulator of apoptotic and inflammatory pathways which are activated during neuro-inflammaging, PD, HD, and AD. Indeed, JNK is involved in inflammatory responses in astrocytes and in primary glial cells. JNK is activated by TNF, IL-1, UV light, and heat shock. Moreover, JNK activation in the brain is associated with intracellular Aβ accumulation and neuronal death in AD patients. It has been also reported that JNKs activity is involved in the regulation of neuronal survival via the maintenance of mitochondrial homeostasis [79–81]. Finally, evolving evidence suggests that p38 plays specific roles in inflammation, cell death, and senescence. Disruption of p38 signaling in neural cells contributes to the pathogenesis of many neurodegenerative diseases including AD, HD, and PD [82]. p38 has been shown to be essential for the maintenance of dopaminergic neurons and oxidative stress and p38 signalling cascade regulate pro and anti-apoptotic phenotypes of dopaminergic neurons [83]. Several studies have investigated the function of p38 in the context of neuroinflammation. Astrocytic p38 signalling is activated by inflammatory cytokines such as IL-1β and TNF-α. It has also been found that p38 in AD is one of the main kinases responsible for excessive tau phosphorylation.

Moreover, accumulation of Aβ plaques is partially mediated through misregulated activity of p38 cascade [84]. Inhibitors of p38 confirm that an elevated activation of this pathway is a critical contributor to neuronal damage and neuroinflammation [85].

### Sirtuin-FoxO longevity pathway

Sirtuins (SIRTs) are members of the class III histone deacetylases, and so far seven sirtuin genes (sirtuins 1–7) have been characterized in mammals [86]. A large number of studies have shown that SIRT1 can alter the fate of a neuron promoting cell survival against stress. SIRT1 appears to be a crucial component for neuronal plasticity and cognitive functions because it is also involved in dendritic and axonal growth. In the brain, SIRT1 regulates numerous neuroprotective functions, including antioxidant and anti-inflammatory response, antiapoptosis, and mitochondrial biogenesis [87, 88]. An emerging target of the sirtuins is the family of FoxO transcription factors [89]. The mammalian FoxOs are composed of four members: FoxO1, FoxO3, FoxO4, and FoxO6. Temporal and tissue specific differences in expression can be observed with FoxO1 and FoxO3 almost ubiquitously expressed, FoxO4 highly expressed in kidney, colorectal and muscle tissue and FoxO6 mainly expressed in the liver and brain. FoxOs are involved in a myriad of cellular processes and programs including energy metabolism, cell cycle regulation, apoptosis, autophagy, immunity, inflammation, resistance to oxidative stress, stem cell maintenance and appear to play a conserved “prolongevity” role observed in worms through to human beings [90]. FoxOs also modulate key aspects of stress response and survival pathways in neurons [91]. Therefore, FoxOs have dual roles in survival and cell death and gene-specific contexts determine the effects of FoxOs on gene expression. Although the effects of SIRT1 on the FoxO target genes are complex, FoxOs can recognize and bind to different sites within the SIRT1 promoter region to induce transcription. SIRT1 activates several members of the FoxO family. Notably, SIRT1 positively controls FoxO transcription by shifting FoxO-dependent response away from cell death and toward stress resistance, particularly in the case of oxidative stress [92]. Three FoxO isoforms are commonly expressed in the brain. FoxO1 is strongly expressed in neuronal subsets of the hippocampus, whereas FoxO6 is highly enriched and expressed throughout the entire adult hippocampus, and appears important for memory consolidation and neuronal connectivity [93]. Of the isoforms, FoxO3 is the most diffusely expressed in the brain and has been shown to be strongly associated with human longevity including a reduced risk for cognitive decline with age for those who possess the protective “G” allele. [94]. SIRT1 deacetylates FoxO3, reducing FoxO-mediated apoptosis but potentiates FoxO-induced cell cycle arrest [95]. SIRT1 and FoxO3 form a complex in response to oxidative stress, and the sites of FoxO3 that appear to be deacetylated by SIRT1 are K242, K245, and K262 [96, 97]. Therefore, the effect of acetylation on FoxO3 has a dual role on function because SIRT1 can increase the ability of FoxO3 to induce cell cycle arrest and resistance to oxidative stress but can also inhibit FoxO3 to induce cell death [89, 98]. Furthermore, the SIRT1-FoxO3 network is associated with the induction of DNA repair proteins. In the context of behavioural adaptations to cocaine, new findings have demonstrated that the induction of SIRT1 deacetylates and activates FoxO3, which then induces many gene targets involved in DNA repair in the brain [99]. Another SIRT widely expressed in neurons and astrocytes is SIRT3. It has been shown to have a major involvement in ROS regulation, deacetylating FoxO3 with consequent expression of CAT and mnSOD [100, 101]. Recently, Rangarajan et al. reported for the first time that SIRT3 expression is induced in activated microglia in vivo and activates antioxidant genes through nuclear translocation of FoxO3 [102].

## Neuroprotective activity of phytochemicals against neuro-inflammaging

**Fig. 3:**
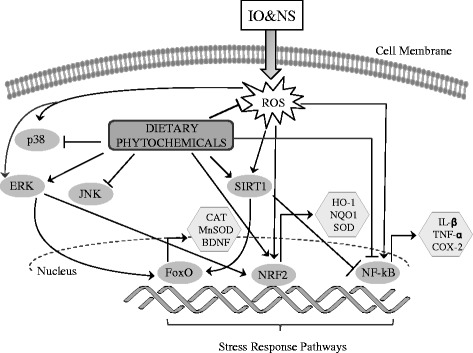
The main intracellular targets involved in the neuroprotective effects of phytochemicals. Most of the phytochemicals with neuroprotective activity seem to converge in the modulation of stress response pathways. For example, phytochemicals can interact directly with Nrf2, allowing the expression of phase II detoxifier genes. The kinases p38, ERK and JNK are also modulated by phytochemicals, regulating both survival and death pathways in response to different stresses. Activation of SIRT1 can regulate FoxO, which modulate genes that encode antioxidant and other stress-response proteins. FoxO is also regulated by ERK in response to a variety of stimuli, including IO&NS and phytochemicals. ERK activation often leads to the activation of Nrf2. Phytochemicals and/or activated SIRT1can also inhibit NF-κB, reducing the expression of inflammatory mediators

Dietary phytochemicals may have a profound effect on many aspects of neuro-inflammaging. Several studies have revealed that the neuroprotective activities of phytochemicals typically occur in dose- and time-dependent manner [42, 103]. A variety of mechanisms appear to underlie the neuroprotective action of phytochemicals. Although research has focused predominantly on antioxidant properties of dietary phytochemicals, the ability of these compounds not only has the potency to scavenge free radical effects in the brain but it has also demonstrated that specific phytochemicals target key stress-activated signaling pathways involved in neuroprotection (Fig. 3). Substantial in vitro and in vivo evidence have suggested that many phytochemicals can affect the expression of numerous genes encoding pro-survival proteins, including antioxidant enzymes, neurotrophic or anti-apoptotic factors. In the next sections, we will present the main classes of food phytochemicals and their emerging role as hormetic inducers of neuroprotective pathways relevant for brain aging.

### Curcumin

*Curcuma* species, particularly *C. longa* (turmeric) have been used in Southeast Asian countries for thousands of years as a food preservative and for medical conditions. Among curcuminoids, the main components in *Curcuma* species, curcumin is the most studied and shows a broad range of pharmacological activities [104]. Its immuomodulatory activity is well-documented and in vivo (100 mg/kg) and in vitro (10 μM) studies have also demonstrated that curcumin can protect dopaminergic neurons against microglia-mediated neurotoxicity and reduce brain inflammation in a concentration-dependent manner [105–108]. Recent studies also indicate an epigenetic role of curcumin, which inhibits the expression of pro-inflammatory mediators by affecting histone acetylation of transcription factors and methylation pattern of gene promoters associated with inflammatory response [109]. Curcumin treatment (2–8 μM) inhibits in a dose-dependent manner the activation of microglial cells by diminishing the production of NO and reducing the secretion of IL-1β, IL-6 and TNF-α (5–20 μM) [110, 111]. Moreover, curcumin blocks the LPS-mediated induction of COX2 via inhibition of NF-κB, activator protein 1 (AP1) (2–16 μM), and signal transducers and activators of transcription (STATs) (5 or 10 μM) [112, 113]. DNA-microarray analyses revealed that 20 μM of curcumin has a strong impact on the microglial transcriptome, leading to an anti-inflammatory and neuroprotective phenotype in LPS-triggered microglia [114]. These concentrations may be indicative of clinical efficacy, since curcumin preparations with enhanced bioavailability (delivered orally) can cross the blood–brain barrier and reach therapeutic concentrations up to 3 μM [115, 116]. Moreover, it should be mentioned that peak plasma concentrations (approximately 1.6 μM) in mice were achieved 15 min after the intraperitoneal administration of 100 mg/kg curcumin, followed by brain accumulation within one hour [117]. In an experimental animal model of chronic epilepsy, 60–100 mg of daily curcumin are effective in attenuating glial immunoreactivity with ameliorative effects on cognitive deficits [118]. Curcumin administration (100 mg/kg) to rats under hypoxic conditions attenuated the upregulation of NF-κB, thereby leading to concomitant downregulation of pro-inflammatory cytokine levels (IL-1, IL-2, IL-18 and TNF-α) and cell adhesion molecules (P-selectin and E-selectin) [119]. Also, repeated intrathecal injection of curcumin (50, 100, 200 mg/kg) dose-dependently attenuates glial activation and spinal neuroinflammation in a rat model of monoarthritis [120]. The TLR4 complex is an important mediator of neuroinflammatory events and treatment with curcumin (50, 100, 200 mg/kg) attenuated TLR4-mediated acute activation of microglia/macrophages, pro-inflammatory mediator release and neuronal apoptosis in the injured brain tissue of rats via inhibition of the MyD88/NF-κB signaling cascade [121]. As reviewed elsewhere, curcumin is also a Nrf2 inducer that upregulates antioxidant defence mechanisms [15]. Recent cell and animal studies have shown that the neuroprotective effects of curcumin (approximately at concentrations of 5 to 25 μM) involves the Akt/Nrf2 pathway, which is consistent with the fact that Nrf2 participates in the neuroprotective effects of curcumin against oxidative damage [122, 123]. Pretreatment with curcumin (5–30 μM) induces neuroprotective antioxidant effects against hemin-induced neuronal death, regulating the expression of Nrf2, HO-1 and glutathione synthesis [124]. Axon degeneration is mediated by microglial MyD88/p38 MAPK signalling and JNK phosphorylation. A new quantitative approach for monitoring axon degeneration found that curcumin (10 μM) protects axons from degeneration during neuroinflammation, inhibiting axonal JNK phosphorylation [125]. The neuroprotective action of curcumin also involves the modulation of SIRT1. Recent observations indicate that SIRT1 signaling activation is associated with the neuroprotective effect of curcumin and preatreatment of curcumin (50 mg/kg) attenuated inflammation, apoptosis, and mitochondrial dysfunction in a rat model of ischemic brain [126]. Curcumin (5–10 μM) was also effective in inducing FoxO3a activity in monocytes/macrophages, suggesting a potential protective mechanism against oxidative damage in the inflammatory cells of the vascular system [127].

### Anthocyanins

Anthocyanins are a class of flavonoids consisting of water-soluble colored pigments. Berry fruits with red, blue or purple colors constitute one of the most important sources of dietary anthocyanins [128]. These compounds are consumed as part of a normal diet and in the United States the human intake of anthocyanins has been approximately estimated to be 180–255 mg/day. Anthocyanins after ingestion reach the circulatory system within 0.25–2 h [129]. Several studies have indicated that anthocyanins from berries may enhance cognitive and motor function during aging due primarily to their antioxidant and neuroprotective properties [130, 131]. Although only a few studies have explored the specific actions of anthocyanins in the context of neuroinflammation, it was recently reported in an experimental model of multiple sclerosis (MS) that anthocyanins (100 mg/kg) suppress the secretion of pro-inflammatory mediators and protect cellular components against oxidative damages induced by demyelination [132]. Moreover, in high-fat-fed animals chronic intake of an anthocyanin extract from blackberry (25 mg/kg) may be capable of preventing the detrimental effects of neuroinflammation with positive effects on synaptogenesis and synaptic plasticity [133]. Anthocyanins protect neuronal cells from pro-oxidant and pro-inflammatory damage via diverse mechanisms, including modulation of Nrf2 and inhibition of NF-κB pathways [134]. For instance, an anthocyanin-rich açaí extract (1 μg/mL) attenuated oxidative stress in rat primary astrocyte cultures through modulation of Nrf2 pathway, thereby restoring the GSH/GSSG ratio and protecting the astrocytic membranes from lipid peroxidation [135]. Furthermore, Aboonabi et al. propose that anthocyanins are efficient inducers of Nrf2 activation and can be considered a good treatment option for inflammation-mediated disorders such as atherosclerosis [136]. Anthocyanins from blueberry, blackberry, and blackcurrant exhibited a similar degree of anti-inflammatory effects and these compounds suppressed the expression and secretion of pro-inflammatory mediators in macrophages by inhibiting nuclear translocation of NF-κB [137]. In another study, the neuroprotective effects of anthocyanins have also been reported in the hippocampus of postnatal rat brain. Anthocyanins (100 mg/kg) inhibited the ethanol-activated expression of JNK, NF-κB, COX-2, as well as attenuated neuronal apoptosis [138]. A blueberry anthocyanin fraction significantly inhibited the LPS-induced production of pro-inflammatory mediators NO, iNOS and COX2 in BV2 microglial cell and this effect was due to the attenuation of NF-κB nuclear translocation [139]. Berries also contain high levels of proanthocyanidins that have neuroprotective effects similar to those of anthocyanins. Findings in primary hippocampal neuronal cells treated with various blueberry fractions (anthocyanins 15 μg/mL; proanthocyanidins 14 μg/ml) and exposed to Aβ_42_ and LPS showed that the major neuroprotective effects of blubbery involve reduction of NF-κB, p38, and JNK [140]. Also bilberry and lingonberry contain high amounts of anthocyanins and proanthocyanidins. At final concentrations of 1–10 μM these molecules exert protective effects against blue light-emitting diode (LED) light-induced retinal photoreceptor cell damage by regulating the activation of NF-κB, p38 MAPK, autophagy and pro-apoptotic proteins [141]. In senescence-accelerated mice prone 8 (SAMP8) mice, blueberry extracts (200 mg/kg) and cyanidin-3-O-galactoside (Cy-3-GAL) (50 mg/kg) may reverse the declines of cognitive and behavioural function, increasing SOD activity, reducing MDA levels in brain tissues and promoting hippocampal ERK expression [142]. Red wine contains a broad spectrum of anthocyanins (Delphinidin-3-glucoside 1.6 mg/L; Petunidin-3-glucoside 2.8 mg/L; Peonidin-3-glucoside 3.8 mg/L; Malvidin-3-glucoside 28.9 mg/L) and a recent study supports the notion that the enhanced myelination after wine treatment in an in vitro mouse model of peripheral nerve system may be induced by SIRTs activation, particularly SIRT1 [143]. Although there are no studies demonstrating that anthocyanins affect the activity of members of the FoxO class of transcription factors, exposure of *Caenorhabditis elegans* to anthocyanin-rich purple wheat (100 μg/mL) leads to a translocation of DAF-16/FoxO to the nucleus, where it stimulates the expression of stress resistance and longevity-related genes [144].

### Flavanols: catechin and epicatechin

Flavanols (also referred to as flavan-3-ols) are one of the largest subclass of flavonoids consisting of monomers, oligomers, and polymers. Catechin and epicatechin are examples of monomeric flavanols and these compounds were found in higher concentrations in cocoa than in other plant-based foods. Flavanol concentrations found in cocoa products are dependent on the cocoa cultivar type, post-harvest handling practices, and manufacturer processing techniques [145]. Fresh and fermented cocoa beans contain approximately 10 % flavanols (100 mg/g) prior to processing, while the cocoa powder consumed by the Kuna Indians contains about 3.6 % flavanols [146]. Although little is known about the pharmacokinetic profiles of flavanols in the brain, catechin and epicatechinin can cross the blood–brain barrier to exert their neuroprotective effects [147]. The process is time‐dependent and epicatechin was found to reach a peak 2–3 h after ingestion and return to baseline value by 6–8 h after consumption of flavanol‐rich chocolate [148]. Thus, it seems plausible that flavanols may beneficially influence the brain, promoting neuroprotection and healthy brain aging. The neuroprotective effects of flavanols are believed to occur through the modulation of several pathways. First, epicatechin can specifically interact with Nrf2-mediated antioxidant response. The administration of epicatechin (30 mg/kg) dose-dependently protects transient ischemia-induced brain injury in both pre- and post-treatment animal studies by activating the Nrf2/HO1 pathway [149]. In addition, it was recently demonstrated in mice that epicatechin (15 mg/kg) protects the brain against injury after traumatic brain injury via Nrf2-dependent and -independent pathways, improving neurologic function, cognitive performance and depression-like behaviours [150]. Pretreatment with 50 or 100 μM of epicatechin also protected primary neurons from oxygen glucose deprivation by increasing neuronal viability and reducing protein oxidation. This effects occurred concomitantly with increased Nrf2-responsive antioxidant protein expression. In the same study, the neuroprotective effect elicited by epicathechin (15 mg/kg) in an animal model of focal brain ischemia was associated with reduced microglia/macrophage activation/recruitment [151]. Further data indicate that catechin exhibits anti-inflammatory effects in LPS-induced BV-2 microglial cells by suppressing the production of pro-inflammatory mediators and attenuating NF-κB activation through regulation of ERK and p38 MAPK pathways [152]. The protective effects of catechin were also demonstrated in experimental models of AD where inflammatory mediators like TNF-α, IL-1β levels and expression of iNOS were significantly attenuated by catechin pretreatment (20 mg/kg) [153]. A recent study revealed that oral administration of cocoa extract (22.9 mg/kg) protects the diabetic retina from glial reaction through SIRT1 activity, providing further evidence that epicatechin can cross the blood retinal barrier and be found in the retinal tissues (4.42 μg of epicatechin/mg of retinal tissue) of animals treated with cocoa [154].

### Oleuropein and hydroxytyrosol

Virgin olive oil (VOO) and extra-virgin olive oil (EVOO) are extracted from olive fruits of *Olea europea* and their health beneficial effects are well established. An extensive literature has demonstrated that these effects can be attributed to many different substances belonging to the phenolic fraction of VOO and EVOO. However, the concentration of these compounds in VOO and EVOO is strongly affected by the particular olive cultivar, by agronomic and environmental factors, and by the extraction and storage conditions [155]. In particular, oleuropein and hydroxytyrosol are the most investigated bioactive phytochemicals and may open new avenues for the development of neuroprotective and/or neurorestorative strategies [156, 157]. Oleuropein and hydroxytyrosol have shown neuroprotective activity by acting against oxidation and inflammation and interfering with amyloid Aβ and tau protein aggregation. Indeed, St-Laurent-Thibault et al. found that these compounds (100 μg/mL) can reduce Aβ-induced toxicity in cultured neuroblastoma cells through involvement of NF-κB signaling [158]. Furthermore, it was recently demonstrated that hydroxytyrosol and oleuropein may be effective as tau aggregation inhibitors at low concentrations (10 μM) in vitro [159]. Mechanistic studies in human neuroblastoma cells reveal that an effective concentration of 5 μM hydroxytyrosol can protect against methylmercury (MeHg)-induced neurotoxicity, causing upregulation of pro-survival proteins including Nrf2 [160]. More interestingly, hydroxytyrosol given to rats at doses of 10 and 50 mg/kg/day improves neurogenesis and cognitive function in prenatally stressed offspring. In this study, hydroxytyrosol supplementation increased transcription factors FoxO1 and FoxO3, as well as phase II enzyme-related proteins, including Nrf2 and HO-1, which may contribute to the decreased oxidative stress and increased mitochondrial function [161]. The neuroprotective effect of hydroxytyrosol was investigated in a model of hypoxia-reoxygenation in rat brain slices after in vitro incubation of this compound or after 7 days of oral treatment with 5 or 10 mg/kg per day. Hydroxytyrosol significantly inhibited the efflux of lactate dehydrogenase (LDH), a marker of brain cell death. Other well-known antioxidants such as vitamin E and N-acetyl-cysteine had no neuroprotective effect in this experimental model [162]. Further observations demonstrate that 100 μM of phenolic compounds (tyrosol, hydroxytyrosol, and oleuropein) from olive oil can inhibit the effect of the chronic inflammatory microenvironment on glioblastoma through regulation of TNF-α, COX-2, JNK, ERK and NF-κB [163]. Hydroxytyrosol also improves neuronal survival, mitochondrial function and reduces oxidative stress in the brain cortex of db/db mice. After 8 weeks of hydroxytyrosol administration at doses of 10 and 50 mg/kg, this compound induced phase II antioxidant systems regulated by Nrf2, activated SIRT1 and the energy-sensing protein network known to regulate mitochondrial function and oxidative stress responses [164].

## Human studies

The link between diet and health is extremely complex and the efficacy of phytochemicals to improve aspects of human brain function during aging is somewhat equivocal. However, over the past decade one of the preventative strategies that has been increasingly accepted is promoting the brain’s endogenous defenses through supplementation with phytochemical-rich foods. The molecular bases for the use of phytochemicals as nutraceutical intervention to prevent cognitive decline are of crucial importance. Animal and in vitro studies have contributed significantly to our understanding, showing a dose-dependent neuroprotective effects. Despite difficulties associated with absorption, bioavailability, and biomarkers, as well as length of the trial and realistic doses, encouraging signs are emerging from intervention and observational studies with improved formulations and appropriate cohorts [165, 166]. Moreover, in order to assess the cause-effect relationship between the consumption of phytochemicals and the beneficial outcome on neuroinflammatory responses, an appropriate study design should always take into account the challenging and controversial regulatory framework of the corresponding authorities worldwide (FDA in USA; EFSA in Europe; FOSHU-Ministry of Health Labour and Welfare in Japan, etc.). In designing human intervention studies and provide high-quality evidence for brain health benefits of phytochemicals, the following factors need to be considered: 1) the phytochemical needs to be sufficiently characterised, 2) optimal physiologic dose, 3) characteristics of targeted populations including their nutritional status, health condition, and genetic background, 4) selection of clinically relevant, sensitive, reproducible, and feasible endpoints, and 5) length of the intervention [167–170]. Moreover, it should be always considered that a phytochemical functional food has a beneficial effect on the health status, thus, the study population should be composed of healthy subjects. However, to date, several human studies have investigated the cognitive enhancing effects of phytochemicals in subjects with both normal cognitive function and people with mild cognitive impairment. The neuroprotective actions of phytochemicals on cognitive function have been attributed to a number of different mechanisms, including antioxidant and anti-inflammatory activities, and increased neurogenesis in the areas of the brain associated with cognition [42]. The lack of specific health claims related to neuroinflammation and brain aging is probably due to the fact that a cluster of clinically relevant markers that reflect the neuroinflammatory state is not well-established. The intake levels and the optimal timing of consumption to prevent age-related cognitive decline in humans have yet to be determined. Despite this, completed human studies on phytochemicals mentioned here are discussed below. The efficacy of curcumin in patients with cognitive decline has been debated, however, Ringman et al. conducted the first 24-week, randomised, double-blind, placebo-controlled study measuring cerebrospinal fluid (CSF) biomarkers and evaluating the efficacy of two dosages of curcumin (2 g/d or 4 g/d) in 36 subjects with mild-to-moderate AD. Bioavailability was reported as a limitation and the results showed no significant differences in cognitive function, in CSF Aβ or tau, between placebo and intervention groups [171]. DiSilvestro et al. evaluated the health promoting effect of curcumin under normal physiological conditions in healthy middle-aged subjects (n 38, 40–60 years). In this placebo-controlled study 80 mg of curcumin (400 mg of Longvida-optimised curcumin) was given orally for 4 weeks, investigating several blood and saliva biomarkers associated with lipids, inflammation, immunity and stress, as well as Aβ levels. Cognitive measures were not included in the study design. Significant changes were shown for several of these markers including increased catalase, NO and antioxidant status, with lowered plasma alanine amino transferase and Aβ levels [172]. Cox et al. randomised 60 healthy adults aged 60–85 using the same curcumin formulation and doses used by DiSilvestro. The authors investigated acute (1 h post dose) and chronic (1-month duration) effects of curcumin intake on mood, blood, and cognition biomarkers. Attention and working memory tasks were improved one hour after administration of curcumin but also after chronic treatment [173]. Age-related cognitive decline is often accompanied by depression. A recent exploratory study provided a partial support of the efficacy of curcumin (500 mg, twice daily) given to 50 participants diagnosed with major depressive disorder. This study demonstrated that curcumin supplementation influences several peripheral biomarkers from salivary, urinary and blood samples and may be associated with its antidepressant mechanisms of action [174].

There is also mounting evidence that dietary supplementation with anthocyanins improve aspects of memory and cognition in older adults. First, supplementation with anthocyanins (300 mg/d) to healthy adults (n 120, 40–74 years) for 3 weeks decreased the plasma concentrations of several NF-κB-regulated pro-inflammatory mediators [175]. Furthermore, a dietary intervention with an anthocyanin-maqui berry extract (486 mg/d) improve oxidative status (Oxidised LDL and F2-isoprostanes) in healthy, overweight, and smoker subjects (n 42 participants, 45–65 years) after 4 weeks of supplementation [176]. Daily consumption of blueberry juice for 12 weeks containing anthocyanins at 877 mg/L improved memory function in older adults with early memory decline (n 9, mean age 76.2) [177]. Anthocyanins comprise 46 % of detected polyphenols in samples of Concord grape juice. Subjects (n 21, mean age 76 years) who received this grape juice for 16 weeks showed increased neural activation in cortical regions along with improved memory function [178]. A recent randomised clinical trial was conducted to assess changes in cognitive function of older adults (n 49, +70 years) with mild-to-moderate dementia after daily consumption of an anthocyanin-rich juice (200 ml/d) over 12 weeks. Secondary outcomes included also blood anti-inflammatory markers (CRP and IL-6). Improvements in verbal fluency, short-term memory and long-term memory were found in the intervention arm but markers of inflammation were not altered [179].

The neurobiological impact of flavanols occurs in two major ways: 1) enhancement of cerebral blood flow throughout the central and peripheral nervous system; 2) interactions with brain signaling cascades increasing expression of neuroprotective and neuromodulatory proteins [180]. To date, evidence for antioxidative and anti-inflammatory properties of flavanols on cognitive decline in human aging is rather limited. However, the known benefits on cerebral blood flow may represent a promising approach in treating cerebrovascular disorders in the elderly. The first dietary intervention study to demonstrate the efficacy of consumption of flavanols on cognitive function was conducted by Desideri et al. in 90 elderly individuals with mild cognitive impairment (mean age, 71 years). In this study, formally known as Cocoa, Cognition, and Aging (CoCoA) study, the subjects were randomised to consume once daily for 8 weeks a drink containing 990 mg (high flavanols), 520 mg (intermediate flavanols), or 45 mg (low flavanols) of cocoa flavanols. The improvement of cognitive performance was recorded in the high- and moderate-flavanol groups, suggesting also a possible role of glucose metabolism in modulating cognitive function [181]. More recently, a parallel-arm of CoCoA study involved 90 elderly individuals without clinical evidence of cognitive dysfunction who were randomly assigned to consume daily for 8 weeks a drink containing 993 mg (high flavanol), 520 mg (intermediate flavanol), or 48 mg (low flavanol) of cocoa flavanols. The results of this study indicate that the regular intake of cocoa flavanols can improve aspects of cognitive performance and these effects appear to be dependent on the amount of flavanols intake [182]. Furthermore, the dentate gyrus (DG) is a key factor in age-related cognitive decline and 3 months of high cocoa flavanol (900-mg daily dose) consumption in healthy 50-69-year-old subjects enhanced DG function in the aging human hippocampal circuit [183]. Finally, high cocoa flavanol drink (494 mg total flavanols; epicatechin 89 mg) improves regional cerebral perfusion in 18 healthy older adults (mean age 61 years), providing additional evidence that cocoa flavanols are associated with benefits for cognitive performance [184]. All these results support the crucial role of flavanols as human dietary supplements in slowing down cognitive decline during brain aging.

Several health claims for olive oil and its derivatives have been assessed in recent years. The only authorized health claim in Europe, relates the impact of olive phenolic compounds on the protection of blood lipids from oxidative stress. “A daily intake of 20 g of olive oil, which contains at least 5 mg of hydroxytyrosol and its derivatives (e.g. oleuropein and tyrosol) provides the expected beneficial effects” [185]. Although the direct cognitive benefits of olive oil require further confirmation and clinical evidence is still lacking, neuropsychological tests in a PREDIMED subcohort of cognitively healthy individuals (n 447, mean age, 66.9 years) demonstrated that adherence to the Mediterranean diet supplemented with EVOO (1 L/week) or nuts (30 g/d) was associated with improved cognitive functions at 4-year follow-up. The authors also measured urinary hydroxytyrosol reporting that the Mediterranean diet plus EVOO group had increased levels of hydroxytyrosol (49.6 μg/L), indicating good adherence with the supplemental foods [186]. However, Crespo et al. recently reported in a double-blind, randomized, placebo-controlled 1-week study that two hydroxytyrosol doses (5 and 25 mg/d) did not modify Phase II enzyme expression in peripheral blood mononuclear cells (PBMCs) of 22 young healthy volunteers [187]. Another study investigated plasma hydroxytyrosol concentration in 62 healthy elderly subjects aged 65–96 years, showing a significant increase after a 6-week daily intake of polyphenol-rich EVOO with high oleuropein contents. The results also show a significant increase of CAT in erythrocytes and a decrease of SOD and glutathione peroxidase activity after EVOO intake [188]. Although several other human studies are still underway, few clinical trials have been conducted thus far. Therefore, it is not possible to make any conclusions concerning the clinical significance of phytochemicals in counteracting neuroinflammation associated with aging and their potential in enhancing cognition. Importantly, even though the concept of hormesis has been explored extensively in terms of its applicability in aging, solid human confirmations are required by future trials.

## Conclusions

Current nutrition recommendations are also directed to prevent neurodegenerative pathologies. Recent evidence suggests that dietary phytochemicals may be particularly attractive for preventing dementia and geriatric cognitive disorders. Brain aging predisposes to neurodegeneration via several mechanisms that in part converge on dysregulation of redox-state and inflammatory pathways. From the topics discussed above, light and shade rise on the effects of phytochemicals on human brain health. However, even though experimental findings have not always translated to a definitive clinical effect, the antioxidant and anti-inflammatory properties of phytochemicals have been widely accepted. Despite the abundance of the literature in this field, a clearer understanding of the mechanisms of action of phytochemicals as modulators of cell signalling pathways involved in neuro-inflammaging play a pivotal role for the evaluation of these molecules in short-term or long-term nutritional intervention trials. Furthermore, the preventive effects of phytochemicals on the onset of neurodegenerative conditions still needs to be critically explored. Many dietary supplements containing phytochemicals are already commercially available and marketed to prevent or ameliorate specific diseases, including age-related cognitive decline. The majority of these products are not substantiated by solid scientific evidence and have not yet been approved by the EFSA and/or FDA. For instance, it is unclear the concentrations of phytochemicals that enter the bloodstream and cross the blood-brain barrier. Although delivery systems, such as nanoparticles, might represent a successful strategy for drug delivery into CNS, the bioavailability continues to be highlighted as a major concern in human intervention studies. The quality of the compounds is another major source of variability and conflicting results. A further challenge is to understand whether dietary phytochemicals have appropriate effects on specific epigenetic mechanisms in specific genes or sets of genes. Brain aging is indeed associated with substantial changes in epigenetic profiles and several preclinical studies have revealed that bioactive phytochemicals play an important role in the modulation of overall epigenetic modifications (histone modifications, DNA methylations and microRNA). Another limitation to the clinical application of these compounds is the lack of knowledge on questions concerning the complex metabolic fate of dietary phytochemicals, the role of the gut microbiota in the bioconversion of phytochemicals, and whether the bacterial transformations produce metabolites with increased biological activity. Future studies addressing these issues are needed. Observational studies and dietary intervention trials in large cohorts of healthy subjects are essential to evaluate whether these phytochemicals can help to prevent age-related neurodegenerative disorders.
